# Update on the management of anticoagulated and antiaggregated patients in dental practice: Literature review

**DOI:** 10.4317/jced.58586

**Published:** 2021-09-01

**Authors:** Jesús Costa-Tort, Verónica Schiavo-Di Flaviano, Beatriz González-Navarro, Enric Jané-Salas, Albert Estrugo-Devesa, José López-López

**Affiliations:** 1 DDS, Master’s student. School of Dentistry, University of Barcelona. University Campus of Bellvitge, Barcelona, Spain; 2 DDS, Master’s degree. School of Dentistry, University of Barcelona. University Campus of Bellvitge, Barcelona, Spain; 3 DDS, Professor of Master’s degree. School of Dentistry, University of Barcelona. University Campus of Bellvitge, Barcelona, Spain; 4 PhD, DDS, MD. Professor of Oral Pathology. Department of Odontostomatology. Faculty of Medicine and Health Sciences (Dentistry), University of Barcelona. University Campus of Bellvitge, Barcelona, Spain / Oral Health and Masticatory System Group (Bellvitge Biomedical Research Institute) IDIBELL, University of Barcelona, L’Hospitalet de Llobregat, Barcelona, Spain; 5 DDS, MD, PhD, Department of Odontoestomatology. Faculty of Medicine and Health Sciences (School of Dentistry), University of Barcelona. University Campus of Bellvitge, Barcelona, Spain. / Dental Hospital University of Barcelona, (Barcelona University) / Oral Health and Masticatory System Group (Bellvitge Biomedical Research Institute) IDIBELL, Barcelona, Spain

## Abstract

**Background:**

Oral antiplatelet and anticoagulant drugs are widely used in a large number of patients visiting the dentist, and there has been much controversy over the years towards their clinical management. The aim to carry out a literature review in order to develop an update on dental management in anticoagulated and / or anti aggregated patients, focusing on oral surgery.

**Material and Methods:**

A bibliographic search was carried out in PubMed on studies published between January 1, 2018, until December 10, 2020, using the keywords (“antiplatelet” OR “antiplatelets”) AND (“dentistry”), and (“anticoagulant” OR “anticoagulants”) AND (“dentistry”).

**Results:**

The number of studies included in this review was 13, and the number of patients among all of them was 3.497 patients under treatment with some type of antithrombotic drug, who underwent different oral surgery treatments.

**Conclusions:**

There is a low risk of peri- and postoperative bleeding events during basic oral surgery treatments in antiplatelet or anticoagulated patients, which can be easily managed through the use of local haemostatic measures.

** Key words:**Oral surgery, antiplatelet drugs, anticoagulant drugs, dental treatment.

## Introduction

The number of anticoagulated patients is increasing worldwide every year, representing approximately 1,9% of the population in Spain, since it is known that today there are between 800.000 and 1.000.000 patients in Spain receiving chronic treatment with some type of anticoagulant drug (1). The use of antiplatelet drugs has also increased, especially as secondary prevention of atherosclerotic disease and because of the higher rate of percutaneous coronary interventions and stent implantation (2). Due to this growth and the fact that in most cases is usually a chronic medication, the number of patients who visit the dentist under antithrombotic treatment is also increasing, being normally older patients and also suffering from systemic pathologies in most of the cases (3).

Even though routine dental treatments are usually low-risk procedures with a little tendency to drawbacks, patients with bleeding disorders, whether they are inherited or acquired, require careful attention when assessing the risk of bleeding; hence a correct surgical management by the professional can avoid these possible adversities (3). It is estimated that approximately 1% of the general population has some type of congenital bleeding disorder, so it is not uncommon to manage this type of patient at the dental practice. (4). For example, treatments for cardiovascular diseases such as heart valve replacement, venous thromboembolism, and especially atrial fibrillation have become something usual, leading to millions of patients worldwide to whom it is prescribed some kind of treatment with anticoagulants or antiplatelet drugs to reduce the risk of thrombosis and the possible sequelae with a high mortality risk (1). As a consequence of this situation, different strategies have been reported when managing antithrombotic therapy at the perioperative time, like the modification in the administration of the antithrombotic drug which should be considered in order to prevent or to avoid possible haemorrhagic events that may take place during or after oral surgery (2,3).

Before any surgical procedure it is important to establish a proper control of haemostasis, especially in patients with high risk of bleeding, in which greater caution must be exercised and all necessary measures must always be available. Before starting the intervention, the risk of bleeding should be assessed according to the type of intervention to be performed, considering as low risk of bleeding some of the most common treatments in daily practice such dental extractions, root scaling and implant surgery, and as high risk of bleeding the extraction of more than 3 teeth, surgical dental extractions, placement of more than 3 dental implants, bone and tissue grafts, sinus lifting, and bone regeneration techniques.

In cases of low risk of bleeding, altering the dose of the drug is not usually indicated (3). However, in case that one or more surgical interventions are required, which despite being low risk separately can present a greater risk of bleeding if performed simultaneously, as well as in other high risk of bleeding procedures, staging has been considered as a preventive measure to avoid possible bleeding events, such as limiting the number of extractions per visit if multiple extractions are required, or the realization of a conservative flap design when their use is required (3,5).

In these cases in which it is necessary to modify any treatment with antiplatelet or anticoagulants, it is essential to ask the responsible doctor of that treatment, who will be the one in charge for its modification or not. Nevertheless, the dentist must take special care in assessing the risk of thromboembolic events which, although being rare, can lead to severe complications for the patient. Therefore, it is important to act with caution since the risk of thromboembolism increases as the drug is discontinued (6-8).

Some authors have tried to categorize the type of surgical haemorrhage according to the perioperative time when bleeding occurs, being classified as primary when they take place at the time of surgery, secondary when they occur in the next hours after the surgery, and reactive when it takes place up to 2 weeks after surgery, being normally as a consequence of infectious processes or persistent inflammation (3,6,9-11).

The aim of this review is to assess the published literature in order to establish an update on dental management in anticoagulated and / or anti aggregated patients, focusing on oral surgery.

## Material and Methods

A bibliographic search was carried out in PubMed on studies published between January 1, 2018, until December 10, 2020, using the filters available in the mentioned database, marking the fields “humans” and “custom range” on the date of publication, selecting the range between the aforementioned years and using the following keywords: (“antiplatelet” OR “antiplatelets”) AND (“dentistry”); (“anticoagulant” OR “anticoagulants”) AND (“dentistry”) 

The inclusion criteria covered all articles about anticoagulants and / or antiplatelet drugs in dentistry, published between the mentioned dates, carried out in human patients, and written in English and Spanish. On the other hand the exclusion criteria covered articles on animal studies, those which did not belong to dental practice or did not deal with antithrombotic drugs in relation to oral surgery and those which were not case-control studies, cohort studies or clinical trials.

Any disagreement was resolved through discussion and consensus between JCT and VSDF. In case a third opinion was required or stalemate, BGN, AED and EJS were consulted to sort out any discrepancy.

## Results

**Figure 1:**
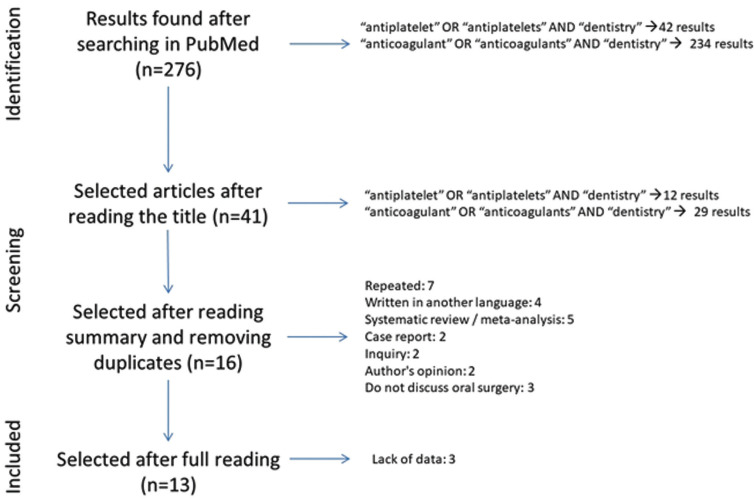
Flowchart.

Using our search strategy, a total of 276 results were obtained. Of these, 42 corresponded to those found using the keywords “antiplatelet dentistry”, and 234 corresponded to those found using the keywords “anticoagulant dentistry”. After reading the titles, 12 of the 42 results obtained using the keywords “antiplatelet dentistry” and 29 of the 234 obtained using the keywords “anticoagulant dentistry” were selected (Fig. 1). The difference between the number of articles found and the number of selected in each group is due to the fact that in the search using the keywords “anticoagulant dentistry”, the same results were also obtained in the previous search using the keywords “antiplatelet dentistry”, in addition to a large number of studies of antithrombotic drugs in relation to other systemic conditions, general surgery which did not concern to dental setting, haematology and cardiovascular medicine, tests and methods for assessment in anticoagulation and antiaggregation, among others.

Of the 41 articles selected based on the title, 7 were discarded for being duplicates, 4 for being written in a different language than those previously established in the inclusion criteria, 5 for being systematic reviews, 2 case reports, 2 surveys, 2 author opinions, and 3 for not dealing with oral surgery. Subsequently, the remaining 16 studies were read in full text and other 2 were excluded; one of them was based on platelet aggregometry as an *in vitro* test to predict the risk of bleeding and for screening the efficacy on platelet inhibition, evaluating its usefulness in general surgery. The other one dealt with the platelet reactivity index, platelet function analysis, and the realization of a visual analogue scale as a method to measure the bleeding index in patients under antiaggregation who received dental implants (Fig. 1).

Finally, the number of studies included in this review was 13: 9 of them were designed retrospectively (3,5,7-11,14,16), 3 were carried out prospectively (12,13,15), and 1 did not specify the type of study (6) (Table 1). Each study was conducted in a single clinical centre and treatments were performed by different operators including specialists, students, residents, and general dentists (Table 2). Among the 13 studies, 2 compared antiplatelet drugs (3,9), 8 compared anticoagulants (5,8,10-15), and 3 compared both antiplatelet and anticoagulant drugs (6,7,16). The number of patients among all the studies added up to a total of 3497 patients who underwent different oral surgery procedures, being dental extractions the most frequent (3,5-16). Other oral surgery treatments performed were the placement of dental implants (3,11,14,16), root scaling (11,16), sinus lift (3,16), cyst enucleation (3), alveoloplasty (3,14), vestibuloplasty (3), biopsies (3), and tori removal (14) (Table 1).

**Table 1:**
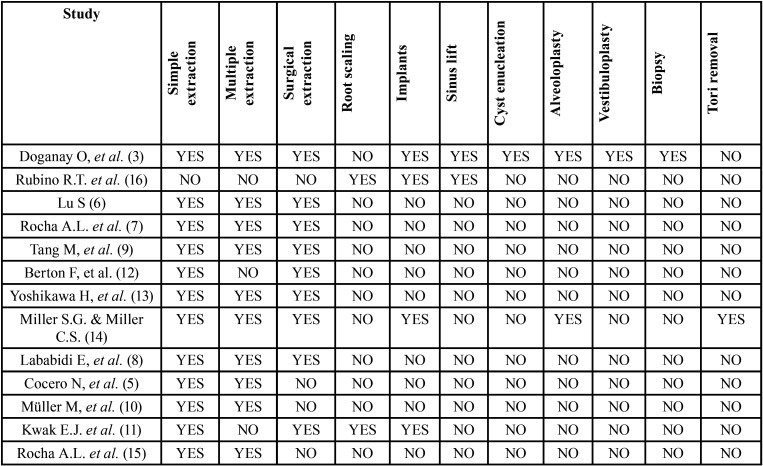
Treatments carried out according to the study.

**Table 2:**
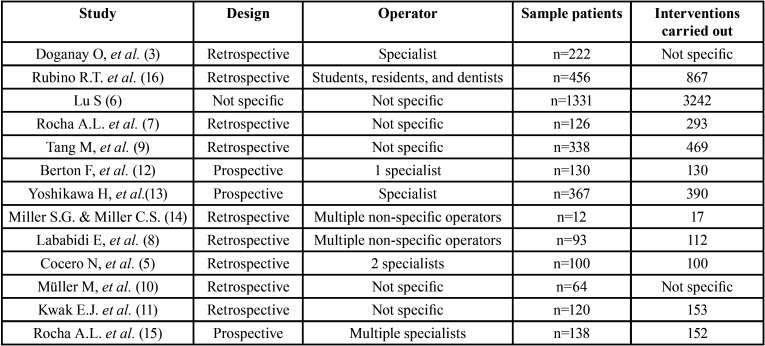
Sample of patients and number of interventions performed.

In one study 3 different antiplatelet drugs were compared and analyzed, in addition to a fourth group with double antiplatelet therapy (3), while in another only ASA (Acetylsalicylic Acid) was compared with clopidogrel (9). In 3 studies (6,7,16), 2 or more antiplatelet drugs were compared with 1 or more anticoagulant drugs, and in other 8 studies only anticoagulant drugs were compared (5,8,10-15), being warfarin the only drug studied in one of them (15). In other 3, only direct-acting oral anticoagulants were analysed (5,11,14), and in the rest direct-acting oral anticoagulants were analyzed and compared with inhibitors of vitamin K, being Warfarin the main drug used in this group (8,10,12,13).

In 6 of the 13 studies, antithrombotic treatment was not withdrawn or modified in any patient (3,5,7,12,13,15), while in other 5 studies it was reported one group of patients in which the pharmacological treatment was not modified, and another one in which the drug was discontinued (8,9,11,14,16). Only one of the studies compared two groups depending on whether or not antithrombotic treatment was suspended, with a similar number of patients in both groups (11), unlike other 4 studies in which the number of patients was different in both groups (8,9,14,16). On the other hand, 2 studies did not specified whether there was any modification or not in the antithrombotic treatment (6,10) (Table 3).

The most frequent reason for prescribing antithrombotic drugs was atrial fibrillation (5,8,10-14), followed by deep vein thrombosis (15), coronary stent (3), percutaneous coronary intervention (9), atherosclerotic cardiovascular disease (7), and prevention of multiple cardiovascular events (6). Only one study did not determine a specific cause for the prescription of these drugs (16) (Table 3). The most widely used antiaggregant was acetylsalicylic acid (3,6,9,16), and in the case of anticoagulants, warfarin was the most widely used (7,8,12,13,15) (Table 3).

**Table 3:**
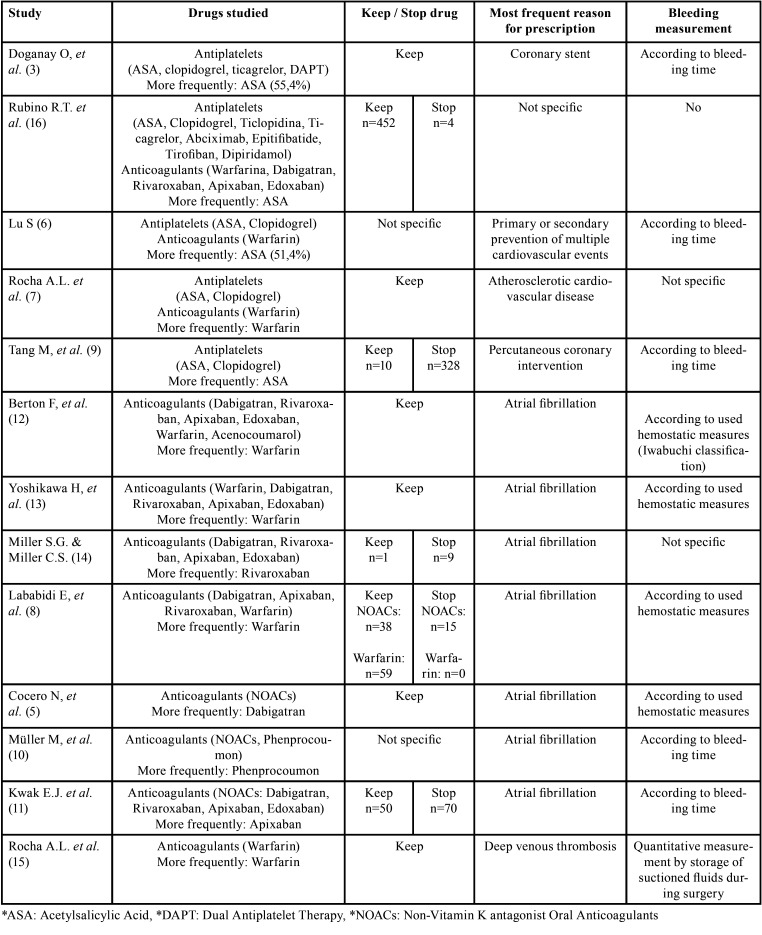
Classification according to the type of pharmacological treatment analysed.

In 5 studies the bleeding index was measured according to the moment in which the haemorrhage occurred, classifying it as perioperative or postoperative (3,6,9-11), while in other 4 it was measured according to the type and number of necessary haemostatic measures used to stop the bleeding (5,8,12,13). Nonetheless, in 1 study it was quantitatively measured by analyzing the volume of blood stored during the intervention using aspiration (13), and in 3 studies no measurement of bleeding was specified (7,14,16) (Table 3). In most of the studies (5-9,11-15), suture and compression techniques with sterile gauze were used as a standard measure in all interventions in order to achieve the wound closure and maintaining a correct control of haemostasis. In cases where simple extractions of erupted teeth were performed, compression with sterile gauze was the only measure used. As additional haemostatic measures, some studies used tranexamic acid (5,6,8,15), ice applications over the treated area (12), and the placement of haemostatic sponges in the surgical wound (5,8,11,13,15). The frequency and appearance of bleeding events in anticoagulated patients showed varied results, ranging from 0% (5,14) to 20% (12), and in the same way in patients receiving antiplatelet therapy, results varied from 1% (6) to 16% (9) (Table 4). Only in 4 out of 13 studies there were statistically significant differences in terms of bleeding events, with a pre-established significant value of *p*≤0.05 (5,7,9,10).

**Table 4:**
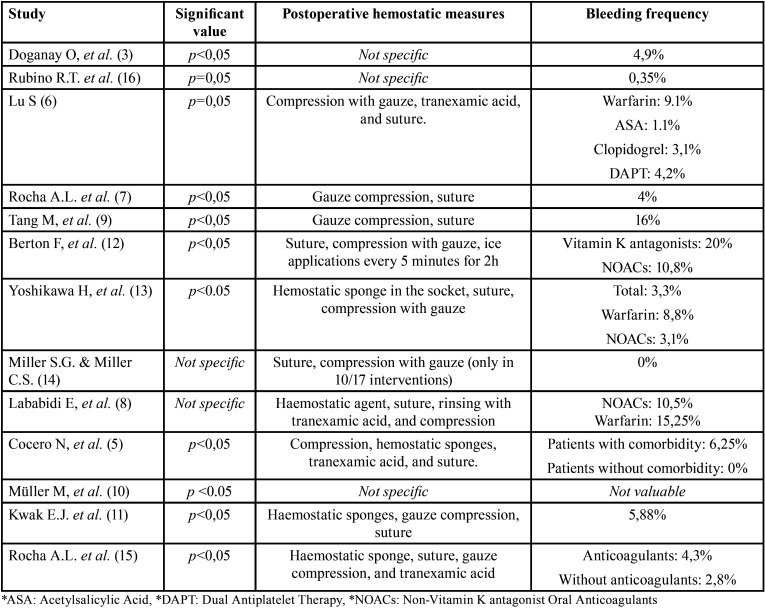
Classification of the haemostatic measures used and the frequency of bleeding events.

## Discussion

Given the need to perform any kind of surgical treatment on a patient undergoing antiplatelet or anticoagulant treatment, the risk of bleeding should be assessed against the risk of thromboembolism which will be assessed by the dentist and the specialist doctor respectively. The specialist is the one who makes the decision to maintain, withdraw, modify, or replace the treatment with the antithrombotic drug, while the dentist must establish all necessary haemostatic measures to control any bleeding that may take place during and after the procedure. According to the results obtained in the studies included in this review, bleeding events that may occur in antiaggregated or anticoagulated patients under oral surgery procedures can be controlled with the use of local haemostatic measures (3,6,11,15,16), which is why it is recommended not to stop antithrombotic treatment in these patients (3,6-8,11-13). On the other hand, in anticoagulated patients with an INR> 3.5, or in patients who require major oral surgery, the option of withdrawing the drug treatment in a period between 0-48h before the intervention may be considered, always consulting the responsible doctor for its modification (7,14). Some international societies and organizations such as the Spanish Society of Oral and Maxillofacial Surgery (SECOM), the American Dental Association (ADA), American College of Cardiology (ACC), or the European Council of Dentists (CED), support these recommendations and criteria for the management of these patients (17). However, according to the study by Kwak *et al*. (11) the authors recommend at least 1 day interruption of the anticoagulant in cases of implant surgery, multiple dental extractions, and deep root scaling, considering the half-life of the drug and renal clearance, although no significant relationship was reported between the duration of anticoagulant discontinuation and the bleeding tendency. However, this disagrees with other authors since although there is a low thromboembolic risk when antiplatelet and anticoagulant drugs are interrupted, it can lead to severe disability or even death, leading discontinuation to a significantly higher morbidity and mortality compared to bleeding events (6-8).

According to the studies included in this review, some of the main factors that increased the incidence of postoperative bleeding were the extraction of more than 2 multiradicular teeth like showed the study by Cocero *et al*. (5), a double antiplatelet therapy as shown in the study by Tang *et al*. (9), and the occurrence of intraoperative bleeding, or interventions performed on patients on dual therapy with ASA and Warfarin compared to those with single Warfarin, accordant to the study by Rocha *et al*. (7). Finally, regarding the comparison between direct-acting oral anticoagulants and vitamin K inhibitors in relation to bleeding events in oral surgery, no significant differences were found between both groups (8,10,13).

There is a lack of consensus when it comes to establishing the guidelines when conducting this type of research, for example regarding the bleeding measurement or the haemostatic measures used in all patients. It would be interesting to establish standard methods to apply to all patients, such as using suture and / or compression techniques with gauze, but without the application of other haemostatic agents that can interfere with the results of bleeding frequency in patients under treatment with any antithrombotic drug. In the same way, it would be useful to have a device which allows us to quantitatively analyze the intraoperative bleeding, like the one used in the study by Rocha *et al*. (15), which consisted of the storage of aspirated fluids in the surgical area, and avoiding the formation of clots in the stored blood by adding 2mL of sodium heparin to the final aspirated solution which were subsequently subtracted from the total volume of aspirated blood. This quantification of perioperative bleeding in oral surgery procedures could help preventing the postoperative bleeding, since the appearance of intraoperative bleeding seems to be related with the predisposition of bleeding events in the next days after surgery (7).

It should be noted that no mention is made on the dosage and posology of the different drugs used by these patients, which could lead to possible limitations in the results. Patients can be grouped according to whether they take the same drug or not, but there is no consideration on the dose or time they have been taking these drugs, in addition to other relevant factors such as the presence of cardiovascular disorders which are neither taken into account.

Due to the wide variety in the methodology and study design used in the different studies included in this review, it was not possible to carry out a meta-analysis of the obtained results, since the different criteria used as well as the values, measurements and factors analysed differ in each of the studies. For example, some of them compare the effects of maintaining / stopping antithrombotic treatment (8,9,11,14,16), while in others the antithrombotic treatment is maintained in all patients equally (3,5,7,12,13,15), and in some others it is not specified whether it was maintained or not (6,10). In some studies like that of Cocero *et al*. (5), Tang *et al*. (9), Kwak *et al*. (11), and Miller & Miller (14), the possible comorbidities reported by the patients and their relationship with bleeding complications were also taken into account, being observed in the one of Tang *et al*. (9) that the incidence of postoperative bleeding was significantly higher in those patients with three or more coexisting conditions, while in the study by Cocero *et al*. (5) the risk of bleeding events observed in patients with comorbidity was not significantly higher than that observed in patients without comorbidity.

According to data obtained from the Spanish Society of Oral and Maxillofacial Surgery, the surgical management of anticoagulated patients has changed significantly in recent years, largely due to the introduction in 1983 of the INR as a method for screening oral anticoagulants therapy, and considering it as an easy standardized method that should be used routinely to control the anticoagulation level of patients treated with these drugs, and require some type of surgical treatment. When planning the surgery, an initial assessment prior to it should be carried out to determine the risk of the mentioned procedure, in which the general condition of the patient, the existence of other haemostasis disorders, the type of intervention to be performed and its expected bleeding degree, the presence of alternatives to surgical treatment, and the patient’s INR at the time of surgery or at most 48 hours before its performance will be taken into account. Regarding the surgery itself it is advisable to assume a series of precautionary measures, like its realization in the morning or early in the afternoon and if possible in the first days of the week, the use of local anaesthetics preferably with vasoconstrictor (with the exception of patients with uncontrolled hypertension or hypothyroidism in whom its indication will be evaluated depending on the case), the use of non-absorbable sutures, and the availability of local haemostatic measures such as gelatine sponges, thrombin, collagen, cyanoacrylate, or oxycellulose.

Regarding the modification or not of anticoagulant treatment, the SECOM, based on the works published by Ardekian *et al*. (18), Blinder *et al*. (19), Campbell (20), and Scully & Wolff (21) offers a series of clinical recommendations which can be obtained on https://www.secomcyc.org/wp-content/uploads/2014/01/cap08.pdf. These are summarized in the following lines:

-For limited oral surgery procedures such as the extraction of 1-3 teeth or taking intraoral biopsies, with an INR <3.5 and without other added risk factors, it is recommended not to modify the anticoagulation.

-In the case of greater surgeries like multiple extractions, lifting flaps, etc., the presence of an INR> 3.5, or the existence of other risk factors, it can be considered the suspension of the anticoagulant two or three days prior to surgery, and the substitution with intravenous heparin in a hospital environment or the use of low molecular weight heparin, and the reinstatement of the anticoagulant the day after the procedure, superimposing both treatments until the desired INR was reached.

Regarding the management in patients under antiplatelet treatment with ASA, the established guideline is:

-At a dose lower than 100mg / day, basic oral surgery procedures such as extractions of 1-3 teeth can be performed without the need of stopping or modifying the antiaggregated state.

-In case of less limited surgeries or in doses higher than 100mg / day, the bleeding time must be determined (time it takes to stop a bleeding caused by a small skin wound), which if it exceeds 20 minutes (normal time <9 minutes), the surgical act will be delayed for 3-7 days.

Regarding the guidelines to follow in the days after the intervention, it is highly recommended to rinse with an antifibrinolytic such as 5% tranexamic acid, 2 minutes / 4 times a day, for 7-10 days. Paracetamol and / or a mild opioid such as codeine are recommended for pain control, avoiding as far as possible prescribing ASA or other NSAIDs due to the increased risk of bleeding they present. Currently, most protocols also recommend the prescription of systemic antibiotics as a prophylactic measure, since the presence of infection is itself an inducing factor of fibrinolysis.

## Conclusions

Based on the results obtained in this review, there is a low risk of peri- and postoperative bleeding events during basic oral surgery treatments in antiplatelet or anticoagulated patients, which can be easily managed through the use of local haemostatic measures. Therefore, it is not recommended to modify the dose or administration of antithrombotic drugs due to the possible adverse effects that could take place which, although infrequent, involve severe consequences for the health and life of the patient. However, in the case of requiring the modification of any treatment with antiplatelets or anticoagulants, it should always be done under the surveillance of the responsible haematologist or specialist doctor.

It is important to know the type of drug used by the patient as well as its possible interactions with other substances that could alter its potency or effect. However, it has not been demonstrated in the studies included in this review that there is a greater predisposition to bleeding according to the type of drug used, or in relation to the presence of associated comorbidities.
